# Athena’s Reply

**Published:** 2015-04-01

**Authors:** Raveenthiran V

**Affiliations:** Professor of Pediatric Surgery, SRM Medical College, Chennai, India

**Dear Sir**

Athena is pleased to know the interest of Prof. Prasad in her analytical review.[1] Rare diseases and controversies go hand in glove because no one has adequate experience to authoritatively affirm or refute claims. Neonatal appendicitis (NA) is no exception to this general rule. The exact pathogenesis of NA is not known. As Athena concluded, it may be related to necrotizing enterocolitis because both the diseases share many risk factors.[2] Dr. Prasad thinks that NA is a local manifestation of systemic inflammatory response syndrome (SIRS).[1] Even if it were true, the cause-effect relationship between the two becomes contentious. Septicemia of NA may cause SIRS and vice versa. Although parietal edema, palpable mass, erythema and tenderness are non-specific of NA, the diagnostic emphasis is on their localization in the right iliac fossa. To conclude, Athena had concluded because she philosophizes that a conclusion is not a conclusion but a beginning.

## Footnotes

**Source of Support:** Nil

**Conflict of Interest:** Author is editor of the journal but he is not involved in decision making of the manuscript.

